# Comparison of catch per unit effort among four minnow trap models in the three-spined stickleback (*Gasterosteus aculeatus*) fishery

**DOI:** 10.1038/srep18548

**Published:** 2015-12-21

**Authors:** Alexandre Budria, Jacquelin DeFaveri, Juha Merilä

**Affiliations:** 1 Ecological Genetics Research Unit, Department Biosciences, University of Helsinki, Helsinki, FI-00014, Finland

## Abstract

Minnow traps are commonly used in the stickleback (Gasterostidae) fishery, but the potential differences in catch per unit effort (CPUE) among different minnow trap models are little studied. We compared the CPUE of four different minnow trap models in field experiments conducted with three-spined sticklebacks (*Gasterosteus aculeatus*). Marked (up to 26 fold) differences in median CPUE among different trap models were observed. Metallic uncoated traps yielded the largest CPUE (2.8 fish/h), followed by metallic black nylon-coated traps (1.3 fish/h). Collapsible canvas traps yielded substantially lower CPUEs (black: 0.7 fish/h; red: 0.1 fish/h) than the metallic traps. Laboratory trials further revealed significant differences in escape probabilities among the different trap models. While the differences in escape probability can explain at least part of the differences in CPUE among the trap models (e.g. high escape rate and low CPUE in red canvas traps), discrepancies between model-specific CPUEs and escape rates suggests that variation in entrance rate also contributes to the differences in CPUE. In general, and in accordance with earlier data on nine-spined stickleback (*Pungitius pungitius*) trapping, the results suggest that uncoated metallic (Gee-type) traps are superior to the other commonly used minnow trap models in stickleback fisheries.

Catch Per Unit Effort (CPUE) is a concept of great conceptual and practical importance in fisheries sciences^1^,^2^,^3^. It is influenced by many factors, both biotic and abiotic, including the fishing gear used^3^. Not surprisingly, effects of different factors and gear types on CPUE in different fisheries have been extensively studied (e.g. ref. 4 and references therein). However, most of these studies have focused on commercially important species, whereas less attention has been placed on species of mainly academic interest (but see refs. 5, 6, 7, 8).

Except for the mainly historical fishery of the three-spined stickleback (*Gasterosteus aculeatus*)^9^,^10^, members of the stickleback family Gasterostidae have not been subject to commercial fisheries. However, they are important model species in behavioural, ecological and evolutionary research^11^,^12^,^13^,^14^. Consequently, a large community of scientists catches sticklebacks from a variety of habitats across their global distribution for diverse research purposes. The methods used in this fishery vary widely, from dip-nets to beach seines and minnow traps. Yet, little research has been conducted in comparing the efficiency of different fishing methods in catching sticklebacks (but see refs. 15, 16, 17, 18).

Earlier studies on stickleback CPUE have focused on comparisons of different trap models and baiting on nine-spined stickleback (*Pungitius pungitius*) catches^15^,^17^,^18^. In the only study focused on three-spined sticklebacks, Merilä^16^ investigated the effect of baiting and trap model on CPUE in the brackish-water environment of the Baltic Sea. While trap model – but not baiting – was found to have a significant influence on CPUE, only two different collapsible canvas trap models were used in that study^16^. Similarly, several studies on nine-spined sticklebacks have verified CPUE differences among different trap models^15^,^17^,^18^. Moreover, the results from these independent studies indirectly suggest that the collapsible canvas traps and the metallic Gee-type^19^ minnow traps might differ in their CPUE. However, no study has yet been conducted to directly compare the efficiency of canvas and metallic trap models in any stickleback fishery.

The main aim of this study was to compare CPUE among four different models of minnow traps (two metallic and two canvas) in the three-spined stickleback fishery. In addition, we also aimed to evaluate whether the observed differences in CPUE could be (at least partly) explainable by differences in the ability of different trap models to retain sticklebacks. Namely, as for any other passive fishing gear, the CPUE of minnow traps depends not only on the probability that fish will encounter and enter into the trap, but also on the probability that they will be retained within the traps until retrieved^4^,^20^. In general, the results should prove as useful guidance for choice of minnow trap model in three-spined stickleback fisheries.

## Results

### Field experiments

A total of 4971 fish (1032 males, 3939 females) were caught during the trapping period. Of these, 3302 were caught from the silver metallic traps, 1330 from the black metallic traps, 330 from the black canvas traps and nine from the red canvas traps.

**Figure 1:**
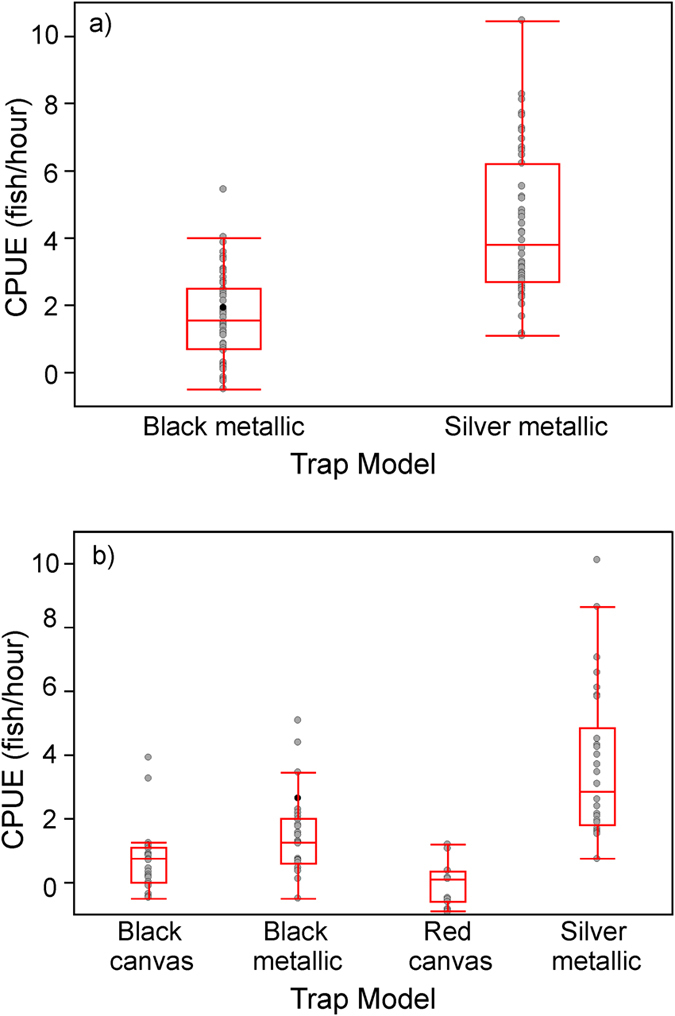
Boxplots for catch per unit effort (CPUE) for different trap models in (a) Experiment 1 and (b) Experiment 2. Boxes depict the 25% and 75% quantiles; vertical line within boxes depicts the median. Whiskers depict 10% and 90% quantiles; dots depict actual data points (data for each replicate site over the six different catches). All values refer to data where site differences have been normalized away.

Experiment 1: CPUE was significantly higher in the silver metallic traps as compared to the black metallic traps (Wilcoxon, χ^2^ = 43.43, P < 0.0001; Fig. 1a). The results were similar when males (χ^2^ = 23.06, P < 0.001) and females (χ^2^ = 45.93, P < 0.001) were analyzed separately. A general mixed linear model confirmed the significant effect of trap type on CPUE (χ^2^ = 41.36, df = 1, P < 0.001), and showed that while CPUE also varied among sites (χ^2^ = 39.25, df = 8, P < 0.001), it was homogenous across different trapping intervals (χ^2^ = 9.57, df = 5, P = 0.08).

Experiment 2: Comparison of all four trap models revealed that the CPUEs differed significantly (Kruskall-Wallis χ^2^ = 68.90, df = 3, P < 0.0001; Fig. 1b). Silver metallic traps yielded the highest median CPUE, followed by the black metallic traps, the black canvas traps and the red canvas traps, respectively, and pairwise Steel-Dwass tests revealed that the mean CPUE differed significantly between all trap types (P ≤ 0.018 in all comparisons). The results were qualitatively similar when males (Kruskal-Wallis, χ^2^ = 65.48, df = 3, P < 0.001) and females (Kruskal-Wallis, χ^2^chi = 67.38, df = 3, P < 0.001) were analyzed separately, except that in pairwise comparisons there was no difference in mean CPUE between the black and red canvas traps for males (Steel-Dwaas, P = 0.24), and between the black metallic and black canvas traps for females (Steel-Dwaas, P = 0.053). As in Experiment 1, a general mixed linear model fitted to this data confirmed the significant effect of trap type on CPUE (χ^2^ = 131.26, df = 3, P < 0.001). CPUE also varied among sites (χ^2^ = 13.87, df = 4, P = 0.008), but was homogenous across different trapping intervals (χ^2^ = 1.76, df = 5, P = 0.88).

### Laboratory experiments

**Figure 2:**
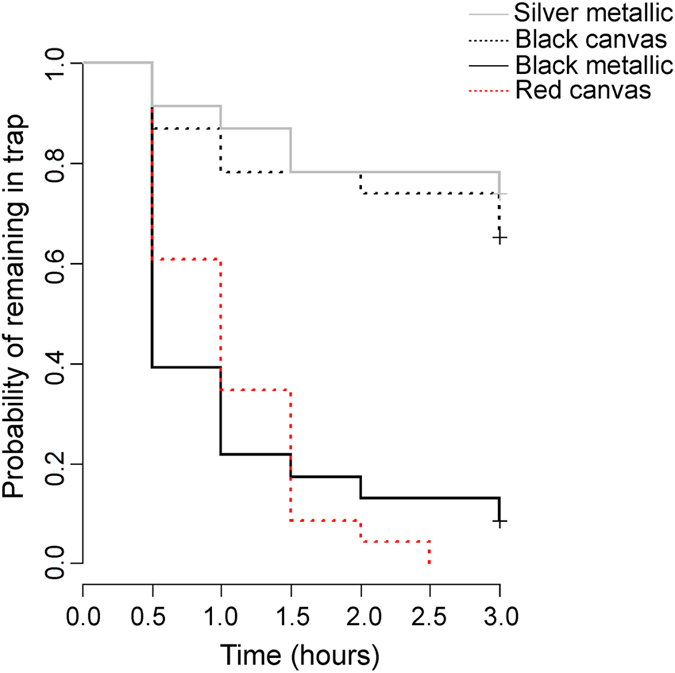
Probability of fish remaining in trap for four different trap models as a function of time-since-initiation of the aquarium experiments. Sample size = 23 for each trap model.

Analysis of escape probability revealed that fish escaped from all trap models with appreciable rates: depending on the trap model, 30 to 100% of the fish had escaped after three hours (Fig. 2). The effect of trap model was highly significant (χ^2^ = 50.6, df = 3, P > 0.001), whereas population of origin (χ^2^ = 4.02, df = 3, P = 0.26), sex (z = −1.616, P = 0.106) and aquaria identity (z = 0.34, p = 0.73) were not. Similar results were obtained when sex was replaced by standard length (z = −0.26, P = 0.8) or weight (z = 0.35, P = 0.73) in the model. Log-rank tests confirmed that escape rates were lowest and very similar (P = 1.00) for the silver metallic and black canvas traps, and far higher and similar (P = 1.00) for the black metallic and red canvas traps (Table 1; Fig. 2). All other pairwise comparisons of trap models across “low” and “high” escape trap models (cf. Fig. 2) were highly significant, even after Bonferroni correction (Table 1).

## Discussion

The most important result of this study was that the four different minnow trap models differed significantly in CPUE, with the silver metallic Gee-type trap having the highest performance of all trap models, by a very large margin. Furthermore, the differences in model-specific CPUEs appeared to depend not only on the probability of sticklebacks to enter the different traps, but also on differences in probability of escaping from them. In the following, the interpretations and implications of these findings are discussed in light of what is previously known about the factors influencing CPUE in stickleback minnow trap fisheries.

The outperformance of the silver metallic Gee-type trap compared to the similarly shaped but differently coated black metallic traps conforms to the results of Merilä *et al.*^18^, who also discovered this to be the case in a freshwater population of nine-spined sticklebacks. Hence, together these results suggest that the traditional silver metallic Gee-trap might generally be a more efficient minnow trap model for stickleback fisheries in a variety of habitats. Our results further suggest that the reason behind the CPUE difference between these two metallic trap models might be in their ability to retain fish once they have entered the trap: the escape probability was significantly higher for the black than for the silver metallic traps. Although the reason for this difference in escape probability remains unresolved, it is noteworthy that in addition to color (black *vs*. silver), the two trap models also differ in wire diameter (black metallic: 1.5 mm; silver metallic: 0.5 mm) and pattern of netting (silver metallic: square-shaped netting; black metallic: diamond-shaped netting; see Fig. 1 in ref. 18). One or all of these factors could generate differences in motivation or ease to escape from the black metallic traps, which could explain their lower CPUE. For example, it is possible that the black color and thicker (and diamond shaped) netting make the black metallic traps more “confined”, allowing fish inside the traps to detect the trap entrances more easily than in the silver metallic traps with different wire characteristics.

The silver metallic traps did not only outperform the black metallic traps, but also both the black and red canvas traps. This is a significant finding, since the performance of metallic and canvas traps has never been directly compared in the stickleback fishery. Several earlier studies have compared the performance of the two canvas trap models, both in three-spined^16^ and nine-spined stickleback fisheries^15^,^17^, and found that the black traps catch more (adult) sticklebacks than the red traps in both species. Hence, the results from earlier studies would encourage the use of black canvas traps over the red ones. However, although the results of this study conform to the earlier findings of the red canvas traps being inferior, this study also revealed that the difference in CPUE between the two canvas trap models is far smaller than that between the metallic and canvas traps. By directly comparing all four trap models, it is clear that the silver metallic trap is the most likely to yield highest CPUEs in stickleback fisheries.

Differences in CPUE among trap models can be ultimately attributed to differences in the rates at which fish encounter, enter and escape from the traps^3^,^21^. As the different trap models in this study were deployed side-by-side, at a distance of only 50 cm apart, we can assume that they were encountered at the same rate. Hence, the observed differences in CPUE should reflect differences in either entry or escape rates. Among the metallic trap models, escape rate was low for the silver trap but high for the black one. Hence, the lower CPUE in the black metallic traps – roughly 50% less than that of the silver traps – might be explainable by their high escape rate. Similarly, the high escape rate from the red canvas traps aligns with their low CPUE: they had the highest escape probability and lowest CPUE of all trap models. This likely explains their consistently low efficiency in catching sticklebacks. However, the black canvas traps had a low CPUE in the field, despite the low escape probability in the lab. This would suggest that in this particular trap model, low entry rate might be the likely explanation for the low CPUE. In other words, the finding that trap model-specific CPUEs and escape probability estimates are not perfectly matching suggests that part of the variation in CPUEs must depend on variation in entry rates. Direct estimates of entry rates would be helpful to establish this inference firmly. In the same vein, although there is clear trap model-specific variation in escape probabilities, it should be kept in mind that these estimates were derived in the laboratory using solitary individuals. Individual and other biotic interactions in the wild might influence escape (and entry) probability in a trap model-specific fashion. Nevertheless, whether caused by variation in entry or escape rates, the differences in CPUE among different trap models were clear and consistent with estimates available from earlier studies^15^,^16^,^17^,^18^.

Finally, although perhaps most often used and discussed in a fisheries context, CPUE also has uses in fundamental ecology and conservation biology by providing information about population abundance^3^,^8^,^22^,^23^. However, CPUE is an accurate index of abundance only if catch efficiency over time and space remains constant and unaffected by other factors^2^,^3^. The finding that the different minnow trap models differ considerably in their efficiency of catching sticklebacks suggests that comparisons of population abundance estimates obtained using different trap models cannot be made reliably – at least not before correcting for differences in catch efficiency among trap models. However, such adjustments may be hard to make if the relative efficiency of the different trap models change in response to variation in biotic and abiotic conditions. For instance, as discussed above, variation in biotic factors influences fish behavior^24^ which in turn can translate to (trap model-specific) variation in entry and escape rates^25^. Hence, given these considerations and the evidence that the silver metallic traps yield consistently higher CPUE estimates than other trap models tested in this study, the silver metallic traps should provide a good standard for population abundance estimation.

In conclusion, the results show that different minnow trap models differ significantly in mean CPUE in the three-spined stickleback fishery, and this variation is likely to be explainable by variation in rates of entry and escape from different trap models. As in the case of earlier reports from the nine-spined stickleback fishery, the silver metallic Gee-traps yielded highest CPUEs. Hence, the silver metallic Gee-traps appear to be the trap model of choice irrespectively of whether the aim is to maximize CPUE or provide an estimate of population abundance.

## Methods

### Field-experiments

Field-experiments were conducted in a brackish-water (average annual salinity 6.03%; 24) bay of Notviken in the southern part of Eckerö, Åland Islands, Finland (ca. 60°11′ 35.66″N, 19°37′06.77″E). Trapping was conducted over five consecutive days (25^th^ to 29^th^ May 2015). All four trap models (see below for description) were deployed at five locations approximately 10 m apart and 1–2 m from the shore. Within each location, traps were randomly set approximately 50 cm apart in dense vegetation, at a depth of 30–80 cm. At an additional four locations, the two models of metallic traps (black and silver) were set in pairs, similar to the abovementioned setup. Hence, a total of nine sets were used: five sets had all four trap models, and four sets had only the two metallic traps. All traps were checked daily. The fish from each trap were counted, sexed according to nuptial coloration of males (i.e. blue eyes, red throat; 12) and released at the site of capture. We note that although this method of sexing stickleback in the field is highly reliable in the breeding season^12^, it is possible that some immature males were mis-classified as females. Soak times between each check varied from 8 to 22 h.

The four different trap models used in the experiments included the galvanized Gee-type metallic minnow trap (Frabill [Jackson, Wisconsin, USA], model # 1279; henceforth: “Silver metallic” trap), the black nylon coated Gee-type metallic minnow trap (Frabill, model # 1271; henceforth: “Black metallic” trap), the collapsible coarse meshed black canvas trap (Promar [Gardena, CA, USA], model TR-503; henceforth “Black canvas” trap), and the collapsible fine-meshed canvas trap (Promar, model TR-501; henceforth “Red canvas” trap). The two metallic and canvas traps are the same as those used in Merilä *et al.*^18^ and Merilä^15^,^16^,^17^, respectively. Detailed trap dimensions and photographs depicting these metallic and canvas trap models can be found from Merilä *et al.*^18^ and Merilä^15^, respectively.

Since the primary aim was to compare the CPUE between the two metallic minnow trap models, both the black and silver metallic traps were used in all nine replicate sets. Being of secondary interest, the two different models of collapsible canvas traps were used only in five of the replicate sets. As a consequence, the study design was not fully crossed and balanced. As such, data were analyzed in two parts. First, the CPUE between the two metallic trap models was compared across all nine sets by leaving out the canvas traps from the five sets where all four trap models were deployed (henceforth: “Experiment 1”). In these analyses, a total of 18 traps (nine traps of each model) were fished over six consecutive checks. Second, the comparison of CPUE between all four trap models was done only for the five sets in which all models were deployed (henceforth: “Experiment 2”). In these analyses, a total of 20 traps (four traps per model) were fished over the six consecutive checks.

### Laboratory experiments

CPUE depends not only on the rate of fish encountering and entering into the traps, but also on the rate of fish escaping from the traps. Therefore, possible differences in the probability that fish will escape from the different trap models were evaluated. These experiments were conducted in the laboratory utilizing two 327.6L tanks (78 × 140 × 30 cm) in the aquaculture facilities of the Department of Biosciences, University of Helsinki, Helsinki, between 17 July and 13 August 2015. The fish used in these experiments were adults caught between May and June 2015 from four different coastal sites in the Baltic and North Seas (55°01′N, 8°26′E; 56°39′N, 9°59′E; 60°27′N, 26°56′E; 65°05′N, 25°23′E). Since the fish from these different populations originated from and were maintained at different salinities, salinity was gradually (over a period of one week) adjusted to 5 parts per thousand for all populations and experimental tanks before starting the experiments.

To estimate escape probability, individual fish were first acclimated to a trap by placing a 50mL tube (containing the fish) within the trap. Sticks were fixed to each end of the tube such that the tube (and hence, the fish) could be maintained in the middle of the trap. After five minutes, the tube was removed, leaving the stickleback in the trap. Starting from this moment, the location of the test individuals was observed every 30 minutes over a period of three hours. If the fish was seen outside of the trap during this period, the trial was terminated and the escape time was noted. Each fish (N = 92) was tested in all trap models once. The sequence of testing the same fish in different trap models was fully randomized.

### Ethics statement

The experiments were approved by the Finnish National Animal Experiment Board under license STH223A, and all used methods were carried out in accordance with approved guidelines. The procedures also adhered to the ‘Guidelines for the treatment of animals in behavioural research and teaching’^26^.

### Statistical analyses

Since the soak time between consecutive trapping sessions varied (see above), the count data was standardized to CPUE by dividing the number of fish caught by the soak time. Hence, the CPUE estimates refer to fish caught per hour.

Repeated measures analysis of variance was not possible since the CPUE estimates were exponentially distributed. Hence, a series of non-parametric tests were used to investigate the effects of trap model, site and gender on CPUE.

For Experiment 1, CPUE was compared between the two metallic trap models that were used in all nine sets, using a Wilcoxon signed-rank test. In order to control for possible set effects, the CPUE data were first normalized by partitioning out the variance due to differences among the sets.

For Experiment 2, CPUE among all four trap models from the five sets (see above) was compared with Kruskall-Wallis analysis. Again, to control for possible set effects, the CPUE data were first normalized by partitioning out the variance due to differences among the sets. Pairwise comparisons among different trap models were compared with Steel-Dwass tests which correct for multiple testing.

In both experiments, all analyses were performed separately for males and females (including possibly some non-breeding males), as well as for the full data. Temporal variation was not incorporated in any of these analyses as there was no temporal variation in CPUE estimates in either of the two experiments, even after normalizing the data first for trap type (Kruskal-Wallis tests; Experiment 1: χ^2^ = 5.55, df = 5, P = 0.35; Experiment 2: χ^2^ = 3.86, df = 5, P = 0.57) or site (Kruskal-Wallis tests; Experiment 1: χ^2^ = 7.45, df = 5, P = 0.19; Experiment 2: χ^2^ = 1.36, df = 5, P = 0.92) effects. Nevertheless, to ensure that our inference is not biased by assumptions about temporal and spatial independence of CPUE estimates, we also fitted generalized linear models for both experiments, where CPUE was modeled as an exponentially distributed response variable (using reciprocal link function), and trap type, set and time interval as fixed effects.

The data on escape probability (reciprocal of probability of fish being trapped) at a given time point was analyzed using Cox- regression^27^, treating trap model, sex, size (standard length), weight, test tank identity and population of origin as factors, and presence or absence of fish within trap as a time dependent binomial response variable. Due to collinearity between sex, size and weight, only one of these three variables at a time was included in the initial model. Model selection was conducted based on Akaike Information Criterion (AIC) and resulted in a model with only one factor: trap model. In this situation, the use of Cox regression is asymptotically equivalent to the use of a log-rank test^28^. Accordingly, log-rank tests^29^ were used to compare pairwise (between trap models) probabilities of escaping, and p-values were adjusted for multiple testing using Bonferroni correction.

Statistical analyses were performed using JMP Pro 11 (SAS Institute, Inc., Cary, NC, USA), except escape probabilities, which were compared using the package “survival” in R^30^.

## Additional Information

**How to cite this article**: Budria, A. *et al.* Comparison of catch per unit effort among four minnow trap models in the three-spined stickleback (*Gasterosteus aculeatus*) fishery. *Sci. Rep.* **5**, 18548; doi: 10.1038/srep18548 (2015).

## Figures and Tables

**Table 1:** Results of log-rank tests of the probabilities of remaining in trap in pairwise comparisons of different trap models.

Comparison	χ^2^	df	P
Black metallic/Black canvas	20.1	1	<0.001 (<0.001)
Black metallic/Red Canvas	0.01	1	1 (0.94)
Black metallic/Silver metallic	24.8	1	<0.001 (<0.001)
Black canvas/Silver metallic	0.4	1	1 (0.53)
Black canvas/Red canvas	26.7	1	<0.001 (<0.001)
Red canvas/Silver metallic	30.9	1	<0.001 (<0.001)

P-values are adjusted for multiple testing with Bonferroni correction (non-adjusted P-values in parentheses). Sample size for each trap model was 23. df = degrees of freedom.
